# Sensory Neuropeptides and their Receptors Participate in Mechano-Regulation of Murine Macrophages

**DOI:** 10.3390/ijms20030503

**Published:** 2019-01-24

**Authors:** Dominique Muschter, Anna-Sophie Beiderbeck, Tanja Späth, Christian Kirschneck, Agnes Schröder, Susanne Grässel

**Affiliations:** 1 Department of Orthopaedic Surgery, Experimental Orthopaedics, Centre for Medical Biotechnology, University of Regensburg, 93053 Regensburg, Germany; anna-sophie.b@arcor.de (A.-S.B.); tanja.spaeth@ukr.de (T.S.); susanne.graessel@ukr.de (S.G.); 2 Department of Orthodontics, University Hospital Regensburg, 93053 Regensburg, Germany; christian.kirschneck@ukr.de (C.K.); agnes.schroeder@ukr.de (A.S.)

**Keywords:** cyclic stretch, mechan-oregulation, murine macrophages, substance P, alpha-calcitonin gene-related peptide, neurokinin receptor, CGRP receptor, destabilized medial meniscus

## Abstract

This study aimed to analyze if the sensory neuropeptide SP (SP) and the neurokinin receptor 1 (NK1R) are involved in macrophage mechano-transduction, similar to chondrocytes, and if alpha-calcitonin gene-related peptide (αCGRP) and the CGRP receptor (CRLR/Ramp1) show comparable activity. Murine RAW264.7 macrophages were subjected to a cyclic stretch for 1–3 days and 4 h/day. Loading and neuropeptide effects were analyzed for gene and protein expression of neuropeptides and their receptors, adhesion, apoptosis, proliferation and ROS activity. Murine bone marrow-derived macrophages (BMM) were isolated after surgical osteoarthritis (OA) induction and proliferation, apoptosis and osteoclastogenesis were analyzed in response to loading. Loading induced NK1R and CRLR/Ramp1 gene expression and altered protein expression in RAW264.7 macrophages. SP protein and mRNA level decreased after loading whereas αCGRP mRNA expression was stabilized. SP reduced adhesion in loaded RAW264.7 macrophages and both neuropeptides initially increased the ROS activity followed by a time-dependent suppression. OA induction sensitized BMM to caspase 3/7 mediated apoptosis after loading. Both sensory neuropeptides, SP and αCGRP, and their receptors are involved in murine macrophage mechano-transduction affecting neuropeptide impact on adhesion and ROS activity. OA induction altered BMM apoptosis in response to loading indicate that OA-associated biomechanical alterations might affect the macrophage population.

## 1. Introduction

As part of the innate immune system, macrophages are one of the most abundant cell types involved in homeostasis by modulating a diverse range of processes including immune response and tissue repair [1]. Macrophages have been shown to respond to a wide variety of endogenous and exogenous stimuli, acquiring an enormous spectrum of different phenotypes [2]. The rather pro-inflammatory M1 phenotype can be considered as one end of the spectrum and the anti-inflammatory, tissue-repairing M2 phenotype as the other end. The high plasticity and activity status of the cells is regulated by the local biomechanical quality of the surrounding tissues displayed by different stiffness values and the direct mechanical impact, leading to adaptation of cells in physiological environments but also in pathophysiological conditions, e.g., cancer and atherosclerosis (reviewed in Reference [3]). Certain tissues like the vasculature, lungs and also bone are especially exposed and adapted to physiological mechanical stimulation but abnormal loading might lead to inflammation and contribute to the disease progression in these tissues [4,5,6].

The musculoskeletal tissues are prime examples for the importance of physiological biomechanical strain to maintain homeostasis. Factors leading to the aberrant loading of, e.g., cartilage, including obesity, malalignment or trauma, are able to induce the development of degenerative pathologies like osteoarthritis (OA). Articular cartilage chondrocytes that experience aberrant load switch their phenotype to a catabolic state and show impaired ability to even respond normally to physiological biomechanical strain, all leading to the degenerative processes observed in OA cartilage [7]. The altered cartilage integrity will most likely change transduction of mechanical forces to the underlying subchondral bone compartment subsequently leading to an aberrant adaptation of bone remodeling processes. Early OA is characterized by an increased bone remodeling rate and a transient loss of bone that turns into densification and sclerosis in long-term OA [8]. Regardless, bones need sufficient mechanical strain to prevent degradation and exercise-derived appropriate loading increases bone strength [9]. In vitro, osteoclasts, the macrophage-derived cells specialized in bone resorption, react to mechanical stress with either an increased resorption activity [10] or a reduction in resorption activity [11,12], depending on the mode and type of the stress applied. Macrophages in the musculoskeletal system not only comprise macrophages resident in bone adjacent tissues, the bone marrow or macrophage-derived osteoclasts. A very important macrophage subset, the osteal macrophage, exists close to bone-forming osteoblasts and is involved in the regulation of osteoblast and osteoclast activity [13]. 

Numerous neurotransmitters and especially neuropeptides derived from sympathetic and sensory peripheral nerve fibers or endogenously produced by local tissue cells are involved in joint physiology and OA-associated degenerative processes. A protective effect in OA has been attributed to sympathetic mediators like vasoactive intestinal peptide (VIP) and pituitary adenylate cyclase-activating peptide (PACAP). Contrarily, the aberrant sprouting of sensory nerves containing the neuropeptides substance P (SP) and alpha-calcitonin gene-related peptide (αCGRP) or increased cellular expression of SP in the subchondral bone and osteophyte remodeling areas was mainly associated with increased pain perception and the induction of a catabolic shift in tissue maintenance (extensively reviewed in Reference [14,15]). 

Interestingly, studies indicate that the endogenous expression of sensory peptides and receptors enables cells to respond to biomechanical stimuli, but no studies exist to our knowledge elucidating their role in pathophysiologies with altered biomechanics, like OA. In mechano-sensitive chondrocytes, studies have shown that the neurokinin receptor 1 (NK1R) is able to sense and respond to mechanical stimulation and react via the endogenous regulation of SP production in chondrocytes [16]. Furthermore, the main mechano-sensing cells of bone, the osteocytes, express the NK1R, but a contribution in mechano-sensation has not been analyzed [17], only indirect findings indicate involvement in osteocyte mechano-signaling [18]. Whether the αCGRP receptor heterodimer calcitonin receptor-like receptor (CRLR)/receptor activity-modifying protein 1 (Ramp1) mediates similar effects in response to αCGRP binding is unknown yet. So far, it was shown in a cyclic loading model of the right ulna using αCGRP^−/−^ mice, that the deletion of the neuropeptide reduced load-mediated mineral apposition responses and new periosteal bone formation [19]. Deletion of both neuropeptides by capsaicin treatment altered the adaptive response to mechanical loading [20]. Primary macrophages derived from the bone marrow express receptors for SP [21] and αCGRP [22] but neuropeptide stimulation also affects the murine macrophage cell line RAW 264.7 [23,24] in vitro. The αCGRP effects on macrophages consist of co-effects with the nerve growth factor (NGF)-directed regulation of major histocompatibility complex class II (MHCII), CD86 and interleukin (IL)-10 expression in human monocytes, as well as lipopolysaccharide (LPS)-driven CD11b and IL-10 expression and a proposed tumor necrosis factor (TNF)α-CGRP regulatory loop in transient receptor potential cation channel subfamily V member 1 (TRPV1)-bearing sensory nerves and immune cells (reviewed in Reference [25]). A well-described αCGRP asset in bone is the inhibition of macrophage differentiation into osteoclasts [26]. SP effects on macrophages comprise the modulation of the M1/M2 macrophage ratio in a diabetic wound healing model in mice favoring the repairing M2 phenotype [27], and the promotion of the M2 phenotype switch in either resting or LPS-stimulated M1-like RAW264.7 cells [28]. SP stimulation enhanced the osteoclast differentiation of bone marrow-derived macrophages in vitro [29]. Both neuropeptides are involved in the regulation of phagocytosis of a monocyte/macrophage cell line [30].

We wanted to elucidate if the sensory neuropeptides SP and αCGRP and their receptors NK1R and CRLR/Ramp1, play a role in the mechano-reception of murine macrophages. Here, we analyzed whether the application of cyclic stretch can induce alterations in the receptor or neuropeptide expression and how cellular reactions like adhesion, proliferation and apoptosis are altered in response to cyclic stretch in these macrophages. Furthermore, we were interested in whether induction of osteoarthritis might alter the reaction of murine bone marrow-derived macrophages (BMM) to neuropeptide stimulation and cyclic stretch. Degenerative joint pathologies like osteoarthritis (OA) might induce altered biomechanical responsiveness in bone tissues but if and how this affects the macrophage population and how sensory neuropeptides participate in this context, is not known to date.

## 2. Results

### 2.1. Mechanical Stretch and Neuropeptide Stimulation Affect Expression of Sensory Neuropeptide Receptor and Endogenous Neuropeptide Expression in RAW264.7 Cells

**Figure 1:**
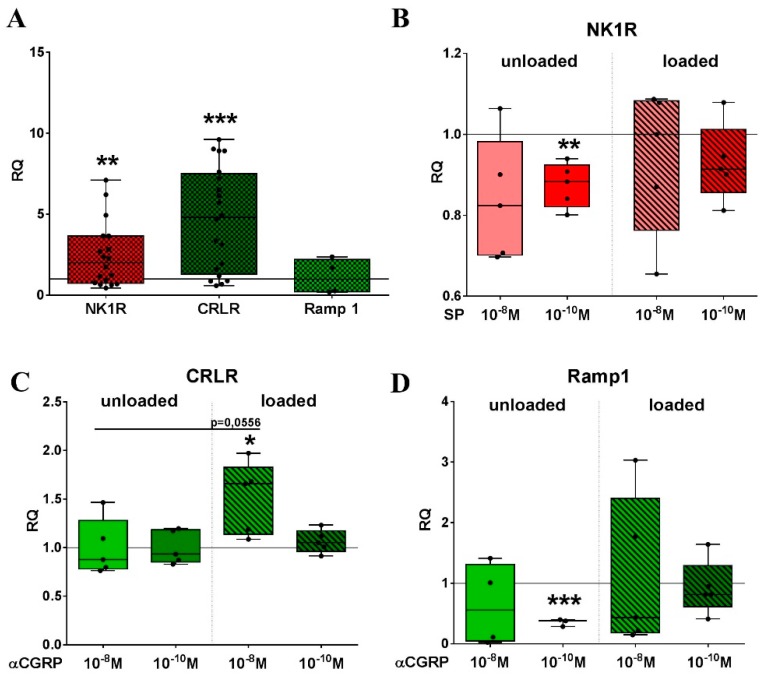
The impact of mechanical loading and neuropeptide stimulation on sensory neuropeptide receptor gene expression of RAW264.7 cells. (**A**) Relative gene expression of *NK1R*, *CRLR* and *Ramp1* after 4 h loading per day on two consecutive days in relation to unloaded cells (= calibrator, RQ = 1) was analyzed using quantitative real-time PCR. Normalizer: *GAPDH*. One sample *t*-test ** *p* < 0.01; *** *p* < 0.001. NK1R, CRLR *n* = 20, Ramp1 *n* = 5; (**B**–**D**) Receptor gene expression was determined after 2 days of loading for 4 h per day in the presence of SP (B, for *NK1R*) or αCGRP (C for *CRLR*, D for *Ramp1*) using quantitative real-time PCR. Normalizer: *GAPDH*. Gene expression of stimulated cells was calibrated to the expression of unstimulated cells (= calibrator, RQ = 1). One sample *t*-test * *p* < 0.05; ** *p* < 0.01; *** *p* < 0.001. *n* = 5. NK1R—neurokinin receptor 1, CRLR—calcitonin receptor-like receptor, Ramp1—receptor activity modifying protein 1, RQ—relative quantification, SP—substance P, PCR—polymerase chain reaction, GAPDH—glyceraldehyde 3-phosphate dehydrogenase, αCGRP—alpha-calcitonin gene-related peptide.

Initial experiments were performed to analyze how mechanical stretch and sensory neuropeptide stimulation affect the expression of the respective neuropeptide receptors and the endogenous peptide production. Gene expression analysis for the *NK1R* as well as the CGRP receptor subunit *CRLR* showed a clear upregulation relative to the gene expression of non-loaded RAW cells after two loading sessions on consecutive days (Figure 1A). The stimulation of RAW cells with 10^−10^ M SP reduced *NK1R* mRNA expression in unloaded cells but not in cells subjected to cyclic stretch (Figure 1B). The mRNA of *CRLR* increased significantly in stretched RAW cells stimulated with 10^−8^ M αCGRP compared to unstimulated cells and to unloaded cells stimulated with 10^−8^ M αCGRP (Figure 1C). *Ramp1* gene expression was reduced by 10^−10^ M αCGRP in unloaded cells, but not after loading (Figure 1D).

**Figure 2:**
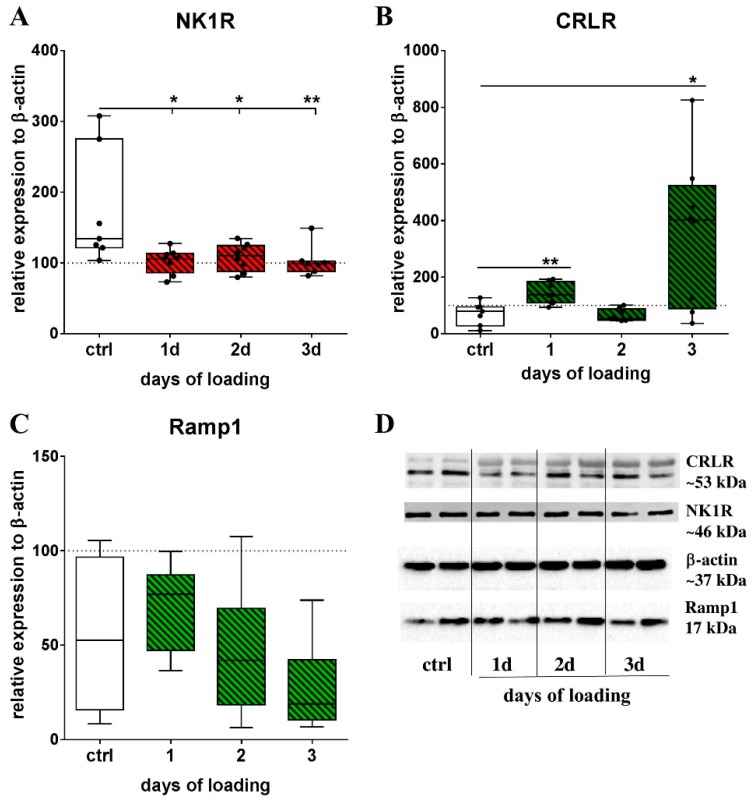
The impact of mechanical loading on sensory neuropeptide receptor protein expression of RAW264.7 cells. Receptor protein expression of NK1R (**A**), CRLR (**B**) and Ramp1 (**C**) was analyzed using the Western Blotting of lysates from cells loaded for 4 h/day on 1, 2 and 3 consecutive days. Expression of β-actin served as endogenous loading control (=100% line). Mann–Whitney test * *p* < 0.05; ** *p* < 0.01; *** *p* < 0.001. *n* = 7–8; (**D**) Representative Western Blot pictures for the CRLR (~53 kDa), NK1R (~46 kDa), Ramp1 (~17 kDa) and β-actin (~37 kDa, endogenous control) of control cells and cells loaded for 1, 2 and 3 consecutive days (presenting 2 lanes for each condition). NK1R—neurokinin receptor 1, CRLR—calcitonin receptor-like receptor, Ramp1—receptor activity modifying protein 1.

Analysis of the protein expression of NK1R, CRLR and Ramp1 by Western Blotting of cell pellet lysates showed a time-dependent effect of mechanical stretch on receptor protein expression. Mechanical loading decreased NK1R protein expression (Figure 2A). The CRLR protein concentration was increased compared to non-loaded cells after 1 and after 3 days (Figure 2B). The Ramp1 protein reduced over the course of 3 days (Figure 2C). Representative pictures of the respective blots for the neuropeptide receptors and the endogenous loading control β-actin are presented in Figure 2D.

**Figure 3:**
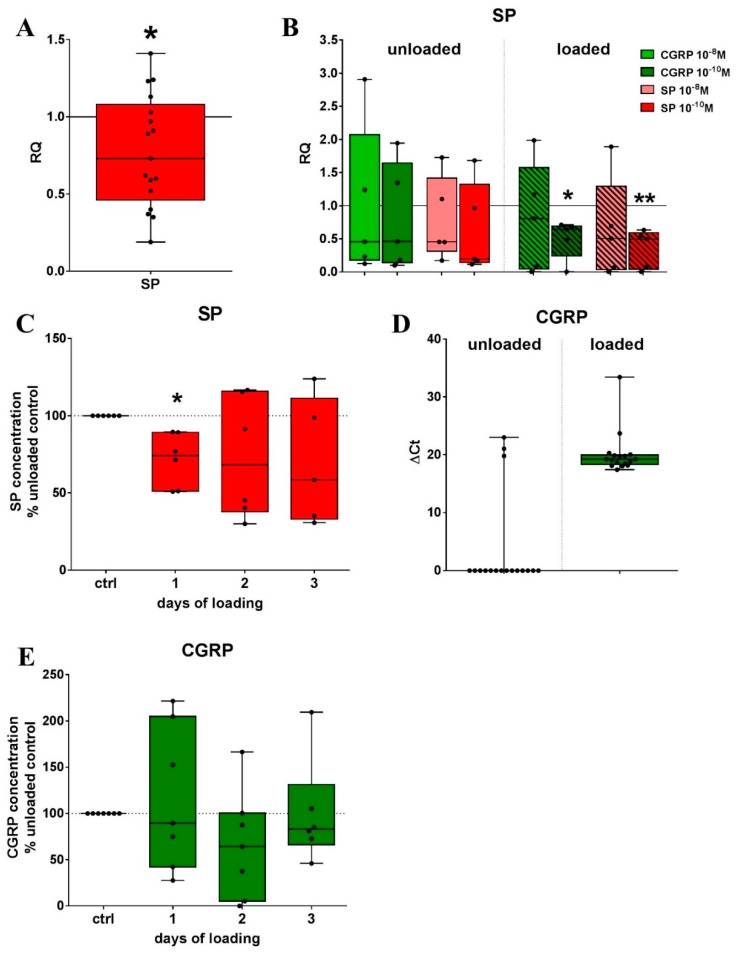
The influence of mechanical loading on gene and protein expression of the sensory neuropeptides SP and αCGRP in RAW264.7 cells. (**A**,**B**) Relative gene expression of *SP* (**A**) and alterations of *SP* expression by stimulation with SP and αCGRP (**B**) after 4-h loading per day on two consecutive days in relation to unloaded cells (calibrator, RQ = 1) was analyzed using quantitative real-time PCR; (**D**) *αCGRP* gene expression is depicted as Δ*C*t of unloaded and loaded cells after 4 h/day stretching on 2 consecutive days. Normalizer: *GAPDH*. One sample *t*-test * *p* < 0.05, ** *p* < 0.01. SP, αCGRP expression *n* = 20, SP expression after stimulation with SP/αCGRP *n* = 5; (**C**,**E**) Protein concentration of SP (**C**) and αCGRP (**E**) was determined in the cell culture supernatants after 4 h/day loading on 1, 2 or 3 days using ELISA. One sample *t*-test * *p* < 0.05. *n* = 7–8. SP—substance P, αCGRP—alpha-calcitonin gene-related peptide, RQ—relative quantification, PCR—polymerase chain reaction, *GAPDH*—*glyceraldehyde 3-phosphate dehydrogenase*.

To evaluate if RAW cells endogenously produce sensory neuropeptides, we analyzed cell culture supernatants after 1, 2 and 3 days of loading by respective ELISA and performed gene expression analysis after 2 days of loading. The mRNA expression of SP in RAW cells was reduced in relation to unloaded cells when load was applied for 4 h each on 2 days (Figure 3A) but was quite low in general (*C*t values 37–39). The stimulation with 10^−10^ M SP or αCGRP during loading significantly decreased SP gene expression in relation to unstimulated cells that were subjected to a mechanical stretch (Figure 3B). Analysis of the cell culture supernatants showed that the secreted SP protein concentration significantly decreased after a single loading session (Figure 3C). 

*αCGRP* gene expression was only occasionally detectable in unloaded cells whereas loading induced a low but detectable expression (Figure 3D). SP and αCGRP stimulation affect *αCGRP* mRNA expression but due to the low expression level, the results are difficult to interpret (Supplementary Figure S1A). The protein concentration of αCGRP in the cell culture supernatant was not affected by the application of mechanical stretch (Figure 3E).

### 2.2. Metabolic Activity of RAW Cells was Affected by Loading and Sensory Neuropeptide Stimulation

Basic cellular functions like adhesion, proliferation and apoptosis were altered after application of mechanical stretch and/or neuropeptide stimulation.

**Figure 4:**
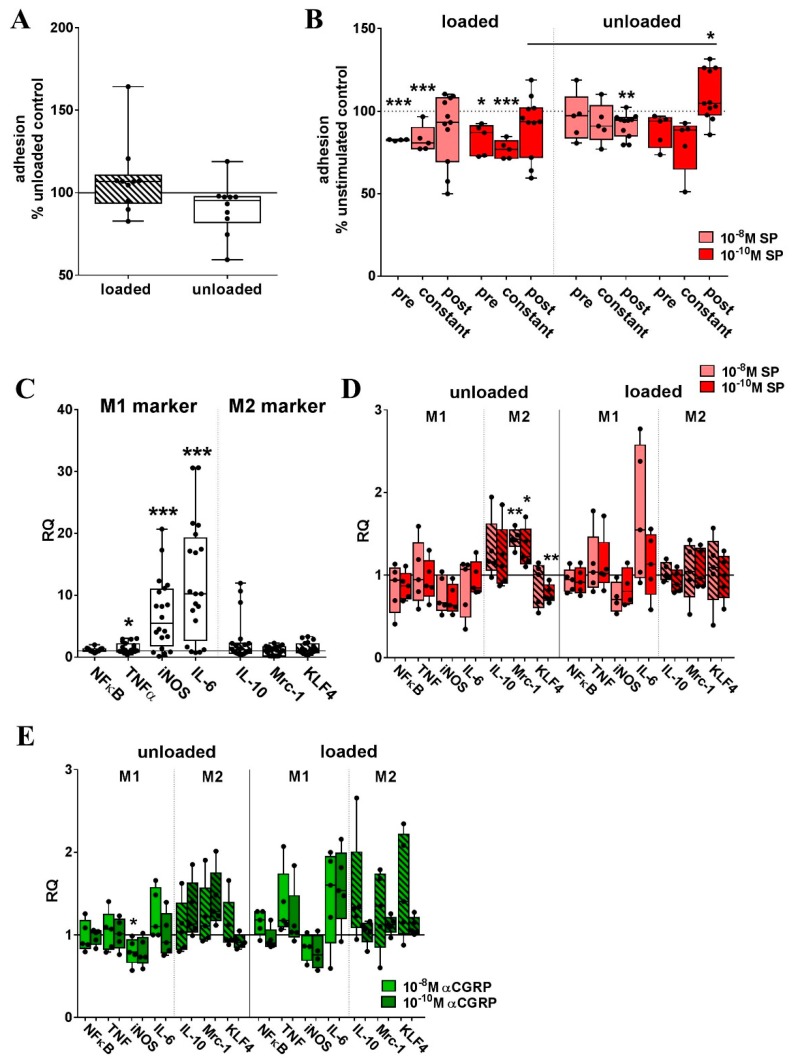
The impact of mechanical loading and sensory neuropeptide stimulation on adhesion and gene expression of macrophage polarization markers of RAW264.7 cells. (**A**) The graph depicts the adhesion of loaded RAW264.7 cells relative to unloaded RAW264.7 cells (set to 100%). One sample *t*-test * *p* < 0.05. *n* = 10; (**B**) shows the impact of stimulation with SP on adhesion of loaded and unloaded RAW264.7 in relation to the respective unstimulated cells (=100%). The graph depicts the effect of stimulation during loading (**pre**), during loading and assay (**constant**) and only during assay procedure (**post**). Pre, constant *n* = 5, post *n* = 11. One sample *t*-test * *p* < 0.05, ** *p*< 0.01; *** *p* < 0.001; (**C**) Analysis of gene expression of M1 and M2 macrophage phenotype markers in loaded RAW264.7 cells in relation to non-loaded cells (calibrator, RQ = 1) analyzed by quantitative real-time PCR; (**D**,**E**) Gene expression of macrophage phenotype markers in loaded and unloaded RAW264.7 cells stimulated with SP (**D**) and αCGRP (**E**) in relation to respective unstimulated cells (calibrator, RQ = 1). Normalizer: *GAPDH*. One sample *t*-test * *p* < 0.05; ** *p* < 0.01; *** *p*< 0.001. unstimulated *n* = 20, stimulated *n* = 5. SP—substance P, αCGRP—alpha- calcitonin gene-related peptide, RQ—relative quantification, PCR—polymerase chain reaction, *GAPDH*—*glyceraldehyde 3-phosphate dehydrogenase*, *NFκB*—*nuclear factor kappa light chain enhancer of activated B cells*, *TNFα*—*tumor necrosis factor alpha*, *iNOS*—*inducible NO synthase*, *IL-6*—*interleukin 6*, *Mrc-1*—*mannose receptor 1*, *KLF4*—*krüppel-like factor 4.*

Loading did not affect the adhesion of RAW cells to plastic compared to unloaded cells (Figure 4A). When cells were stretched in the presence of 10^−8^ M or 10^−10^ M SP, the adhesion capacity was reduced compared to the unstimulated cells. In non-loaded cells, SP only affected adhesion when applied during assay (constant). The adhesion capacity of unloaded cells was reduced by stimulation with 10^−8^ M SP, whereas 10^−10^ M SP increased the adhesion to plastic in relation to unstimulated but also to loaded cells stimulated with 10^−10^ M SP (Figure 4B). αCGRP effects on adhesion were minor, only the stimulation of loaded RAW cells with 10^−10^ M αCGRP reduced the adhesion to plastic (Supplementary Figure S1B).

Additionally, the cyclic stretch did not affect the proliferation of RAW cells (Supplementary Figure S2A). SP stimulation only affected proliferation when applied short term during assay (post). SP suppressed proliferation at a concentration of 10^−8^ M and 10^−10^ M whereas αCGRP had no effect (Supplementary Figure S2B,C).

Caspase 3/7-mediated apoptosis increased over a time course of 24 h in loaded cells compared to non-loaded controls (Supplementary Figure S2D). Additional neuropeptide stimulation induced a high inter-experimental variability without a clear trend (Supplementary Figure S2E,F).

### 2.3. Mechanical Stretch-Induced M1 Phenotypic Marker Genes in RAW264.7 Cells

Different macrophage phenotypes are important for the initiation of an inflammatory reaction or for the initiation of tissue repair processes and the presence of the appropriate phenotype is indispensable for the maintenance of homeostasis. Mechanical strain and neuropeptides are among the factors determining the acquisition of a certain phenotype.

In our experimental design, we observed the upregulation of mRNA for *inducible NO synthase* (*iNOS*), *TNFα* and *IL-6*, characteristic for the M1 phenotype, after two loading sessions (Figure 4C). In non-loaded RAW264.7 cells, stimulation with 10^−8^ M or 10^−10^ M SP significantly increased the expression of the M2 marker *mannose receptor 1* (*Mrc-1*). Another M2 marker gene, *krüppel-like factor 4* (*KLF4*), was significantly decreased by 10^−10^ M SP. In both loaded and unloaded cells, *iNOS* expression was by trend reduced by SP (Figure 4D). We observed only little αCGRP effects on macrophage polarization, except for a trend towards a reduction in *iNOS* mRNA expression that was significant after stimulation with 10^−8^ M αCGRP in unloaded cells (Figure 4E).

### 2.4. Loading in the Presence of Neuropeptides but not Loading Alone Evoked a Strong Increase in ROS Activity in RAW264.7 Cells

Numerous mediators are able to induce reactive oxygen species (ROS) production in macrophages and neutrophils. Here, we elucidated the effect of combined loading and SP or αCGRP stimulation on the ROS production of RAW cells. 

**Figure 5:**
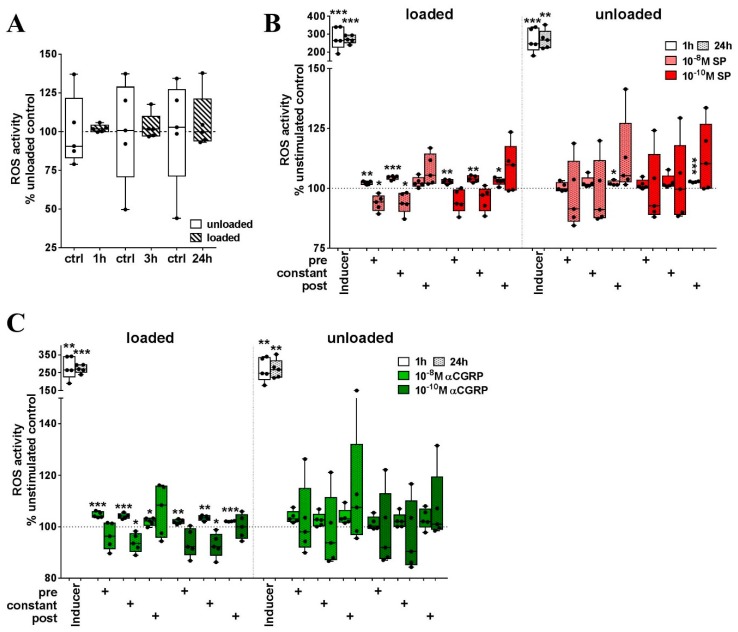
The impact of mechanical loading and sensory neuropeptide stimulation on the production of ROS in RAW264.7 cells. (**A**) ROS activity of loaded cells compared to unloaded cells (=100%) 1, 3 and 24 h after loading; (**B**,**C**) Effect of SP (**B**) and αCGRP (**C**) stimulation on ROS production of loaded and unloaded RAW264.7 in relation to the respective unstimulated cells (=100%) after 1 and 24 h. The graph depicts the effect of stimulation during loading (**pre**), during loading and assay (**constant**) and only during assay procedure (**post**). Effective ROS induction was verified using an inducer that was included in the assay kit (positive control). *n* = 5. One sample *t*-test * *p* < 0.05; ** *p* < 0.01; *** *p* < 0.001. ROS—reactive oxygen species, SP—substance P, αCGRP—alpha-calcitonin gene-related peptide.

Loading alone did not induce ROS production over time (Figure 5A). When cells were stimulated with 10^−8^ M or 10^−10^ M SP during loading, we measured an initial increase in ROS production after 1 h. When stimulated after the loading procedure, 10^−10^ M SP was able to induce the expression of ROS in RAW cells. In unloaded cells, short–term stimulation (only during the assay procedure = post) with high and low SP concentrations induced ROS (Figure 5B). After 24 h, 10^−8^ M SP applied during loading (pre) significantly reduced the amount of ROS in relation to unstimulated, loaded cells (Figure 5B). αCGRP stimulation, regardless of whether it was applied during or after loading, induced a strong initial increase in ROS in loaded RAW cells. Constant αCGRP stimulation of loaded cells significantly reduced cellular ROS content in relation to unstimulated, loaded cells after 24 h. Without loading, αCGRP stimulation was less effective (Figure 5C).

### 2.5. Mechanical Strain and Neuropeptide Stimulation are Regulators of Osteoclast-related Gene Expression

**Figure 6:**
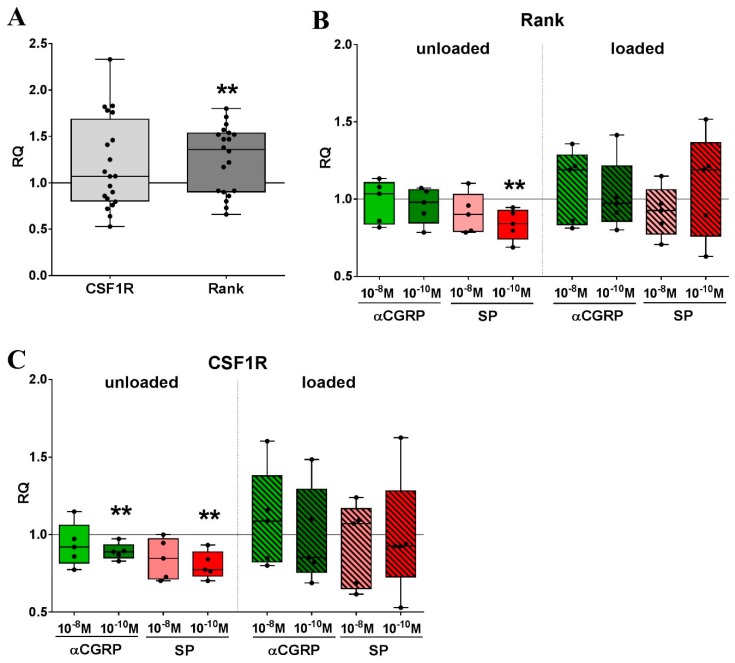
The impact of mechanical loading and sensory neuropeptide stimulation on the gene expression of osteoclast differentiation markers in RAW264.7 cells. (**A**) Analysis of gene expression of the receptor for M-CSF (*CSF1R*) and *Rank* in loaded RAW264.7 cells in relation to non-loaded cells (= calibrator, RQ = 1) analyzed by quantitative real-time PCR; (**B**,**C**) Gene expression of *Rank* (**B**) and *CSF1R* (**C**) in loaded and unloaded RAW264.7 cells stimulated with SP and αCGRP compared to the respective unstimulated cells (calibrator, RQ = 1). Normalizer: GAPDH. One sample *t*-test * *p* < 0.05; ** *p* < 0.01; *** *p* < 0.001. unstimulated *n* = 20, stimulated *n* = 5. *CSF1R*—*colony stimulating factor 1 receptor*, RQ—relative quantification, PCR—polymerase chain reaction, GAPDH—glyceraldehyde 3-phosphate dehydrogenase, SP—substance P, αCGRP—calcitonin gene-related peptide.

Macrophages provide the precursor pool for bone-resorbing osteoclasts in an environment tightly regulated by mechanical influence. Cyclic stretching of murine RAW macrophages induced the upregulation of the *rank receptor* mRNA, the main factor in the regulation of osteoclast differentiation (Figure 6A). In combination with neuropeptide stimulation, loading had no clear effect on the gene expression of *Rank*. Without additional loading, the stimulation with 10^−10^ M SP reduced expression of *Rank* mRNA in relation to unstimulated cells (Figure 6B). Gene expression of the *colony-stimulating factor 1 receptor* (*CSF1R*) was not affected by loading, but 10^−10^ M SP or αCGRP were effective in reducing gene expression of the *CSF1R* in non-loaded RAW264.7 macrophages compared to non-stimulated cells (Figure 6C).

### 2.6. Bone Marrow-derived Macrophages from OA Mice Show Differential Reaction to Loading

We intended to compare the effect of loading and neuropeptide stimulation on apoptosis and the proliferation of BMM derived from mice after osteoarthritis surgery (DMM) compared with Sham-operated animals. The proliferation of BMM from DMM mice 2 and 8 weeks after surgery, was unaffected compared to the unloaded cells and to BMM from Sham animals (Supplementary Figure S3A). Neuropeptide stimulation had no additional effects (Supplementary Figure S3B–E). 

**Figure 7:**
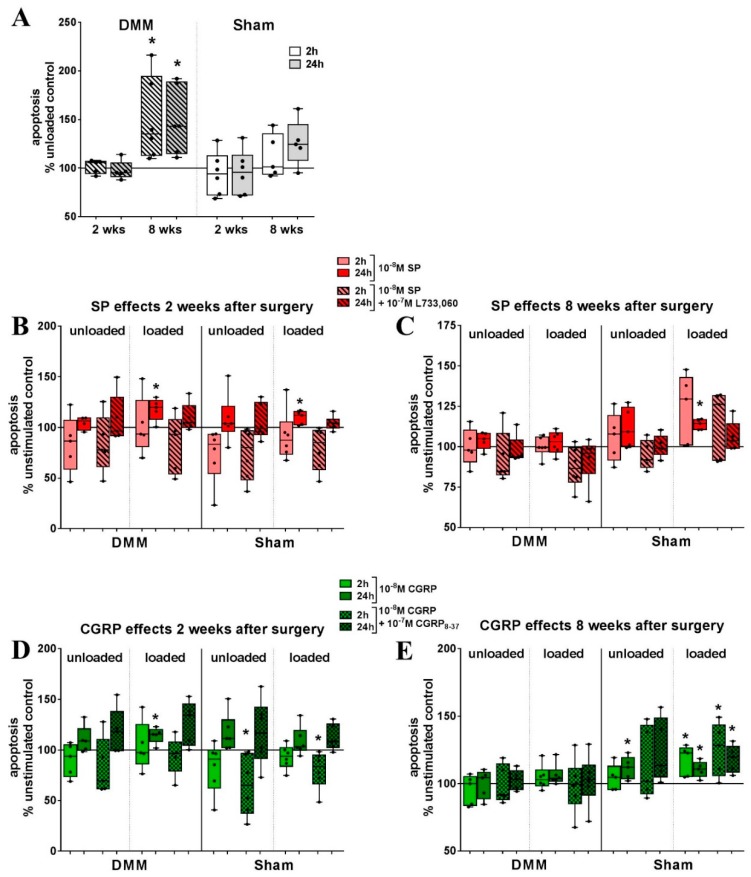
The impact of mechanical loading and sensory neuropeptide stimulation on the apoptosis of BMM from DMM and Sham mice 2 and 8 weeks after surgery. (**A**) Analysis of caspase 3/7 mediated apoptosis of BMM isolated 2 and 8 weeks after DMM or Sham surgery and subjected to 4 h loading per day on 2 consecutive days depicted in relation to the proliferation of unloaded cells (=100%). *n* = 5–6. One sample *t*-test * *p* < 0.05; (**B**–**E**) Effect of stimulation with SP or SP combined with the NK1R antagonist L733,060 (**B**,**C**) or αCGRP and αCGRP combined with the αCGRP receptor antagonist CGRP_8–37_ (**D**,**E**) on apoptosis of BMM isolated 2 (**B**,**D**) and 8 (**C**,**E**) weeks after DMM or Sham surgery in relation to unstimulated cells (=100%). One sample *t*-test * *p* < 0.05. *n* = 5–6. BMM—bone marrow-derived macrophages, DMM—destabilized medial meniscus, SP—substance P, αCGRP—alpha-calcitonin gene-related peptide, NK1R—neurokinin receptor 1.

Isolated 8 weeks after OA induction, BMM from DMM mice were significantly more sensitive to loading-induced caspase 3/7-mediated apoptosis compared to unloaded cells (Figure 7A). In loaded BMM from DMM but also from Sham mice isolated 2 weeks after surgery, SP increased apoptosis after 24 h and was restored to control levels in combination with the NK1R antagonist L733,060. Short-term stimulation with SP (2 h) alone, as well as in combination with L733,060, reduced apoptosis in BMM from Sham animals 2 weeks after surgery, compared to non-stimulated cells. Loaded cells from these mice only showed a reduction of apoptosis when stimulated with the combination of SP and L733,060 (Figure 7B). Apoptosis of BMM, isolated 8 weeks after DMM surgery, was not affected by SP stimulation. In loaded cells from Sham animals, SP increased apoptosis after 2 and 24 h compared to unstimulated and unloaded, SP-stimulated cells (Figure 7C). 

αCGRP effects on BMM from DMM mice 2 weeks after surgery were only apparent after loading. Increased apoptosis was detected after 24 h, when loaded BMM were stimulated with 10^−8^ M αCGRP and the effect was not reduced by co-stimulation with CGRP_8–37_. In unloaded BMM from Sham animals isolated after 2 weeks, αCGRP increased apoptosis after 24 h but not in loaded cells where the combination with CGRP_8–37_ increased apoptosis at this time point (Figure 7D). Isolated 8 weeks after OA induction, apoptosis was increased in loaded BMM in the presence of 10^−8^ M αCGRP. In BMM from Sham animals, the apoptosis of non-loaded cells was increased with 10^−8^ M αCGRP but also in combination with CGRP_8–37_. Loaded Sham BMM were even more sensitive to αCGRP-mediated apoptosis where it increased after 2 h and remained increased after 24 h, also in combination with CGRP_8–37_ (Figure 7E).

BMM from mice isolated 8 weeks after DMM or Sham surgery were subjected to osteoclastogenesis by stimulation with MCSF and Rankl. Loading was applied for 5 days during differentiation. The results are highly variable but by trend, absolute osteoclast numbers seem to be reduced by loading as well as loading in combination with SP and αCGRP stimulation independent from OA induction (Supplementary Figure S4).

## 3. Discussion

**Figure 8:**
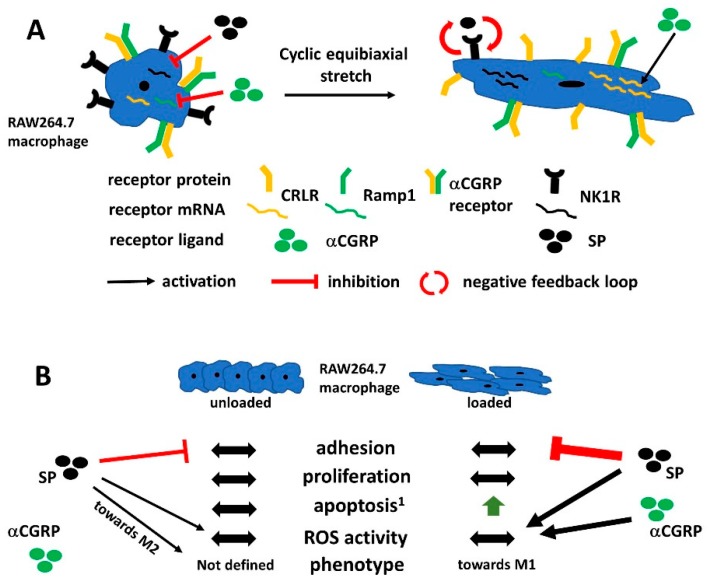
The schematic summary depicting the influence of the cyclic stretch on RAW264.7 macrophage cells in the context of sensory neuropeptide stimulation. (**A**) Physiological stimulation with SP and αCGRP decrease mRNA expression of their respective receptors, *NK1R* and *Ramp1* (an αCGRP-specific subunit of the αCGRP receptor) in unloaded conditions. Cyclic stretch-induced upregulation of mRNA for *CRLR* (unspecific αCGRP receptor subunit) and *NK1R*. αCGRP further upregulated the *CRLR* gene expression resulting in increased CRLR protein expression. Oppositely, albeit upregulation of *NK1R* mRNA, protein expression decreased, possibly by lack of receptor-ligand SP that was also decreased by cyclic stretch. In loaded RAW264.7 cells, the cyclic stretch might induce a negative feedback loop involving the downregulation of the neuropeptide SP and its respective receptor, NK1R; (**B**) Comparison of cellular traits like adhesion, proliferation, apoptosis, ROS activity and the macrophage phenotype in loaded RAW264.7 macrophages to unloaded controls demonstrated a strong induction of apoptosis and a phenotypic change towards the M1 phenotype. In unloaded cells, SP inhibited adhesion, increased ROS activity and induced the expression of M2 macrophage marker genes. αCGRP effects were marginal. Oppositely, cyclic stretch sensitized macrophages to strong inhibition of adhesion by SP as well as a strong increase in cellular ROS activity by stimulation with SP and αCGRP. ^1^ The same increase of apoptosis after loading was observed in bone marrow-derived macrophages from mice that underwent surgical OA induction. αCGRP—alpha calcitonin gene-related peptide, CRLR—calcitonin receptor-like receptor, NK1R—neurokinin receptor 1, Ramp1—receptor activity modifying protein 1, SP—substance P, OA - osteoarthritis.

Macrophages are highly adaptive cells with variable phenotypic plasticity allowing them to respond to different environmental requirements. Exogenous factors influencing macrophage phenotypes include biochemical mediators but also mechanical stimuli. In previous studies from our group, we showed that sensory neuropeptides, especially SP, affect bone cells like osteoclasts and their progenitors derived from the macrophage lineage [21]. Furthermore, cells of the musculoskeletal system are exposed to and regulated by partly high mechanical forces raising the question of how this combination of stimuli would affect macrophages. In the musculoskeletal tissues, except for the articular cartilage, macrophages act as innate immune cells and provide the precursor pool for osteoclasts. They are also known to express functional receptors for SP and CGRP [21,22]. Classical musculoskeletal cell types affected by mechanical load include chondrocytes, tenocytes, osteoblasts or osteoclasts. Millward-Sadler and colleagues previously demonstrated the participation of sensory neuropeptides in mechano-regulation and identified SP and the NK1R as mechano-regulators of chondrocyte behavior in a paracrine and autocrine way [16]. Stretch, applied using the Flexcell system, increased *SP* mRNA expression and decreased *NK1R* mRNA in human tenocytes compared to unloaded tenocytes [31] opposite to our results observed in murine macrophages. In accordance with our data, the stimulation of tenocytes with SP decreased *NK1R* expression, while *SP* mRNA was further reduced by stimulation with picomolar concentrations of SP and CGRP in murine macrophages. To the best of our knowledge, this is the first study identifying the Tachykinin system being involved in macrophage mechano-regulation. In the Atlantic salmon, exercise-induced loading increased SP (mRNA, protein) and NK1R (protein) in osteocytes (only SP) and osteoblasts [18]. Altogether, our observations partly disagree with previous studies presumably due to differences in the type and mode of load, the general reactivity of cells to loading and a complicated interaction of various cell types when mechanical effects are evaluated in vivo. Regarding SP and its receptor NK1R, we propose a negative feedback loop induced by cyclic stretch reducing the protein of both the receptor and ligand (Figure 8A). Additional variation in the responsiveness of the NK1R might be caused by altered cellular receptor localization leading to the activation of alternative signaling pathways. Cell membrane bound G-proteins are known to elicit different responses compared to internalized receptors, which form an endosomal signalosome (reviewed by Cattaruzza et al. [32]). 

The CGRP receptor, composed of the subunits CRLR and Ramp1, has not been described before to be directly mechano-responsive. In monoiodacetate-induced arthritis (MIA) and MMT (medial meniscus transection) rat OA models, αCGRP increased the mechano-sensitivity of joint nociceptors and the mRNA and protein levels of CRLR were upregulated in afferent neurons [33]. We clearly showed that stretch affected the expression of *CRLR* and *αCGRP* in RAW264.7 macrophages with additional upregulation of *CRLR* after αCGRP stimulation under loading (Figure 8A). These findings demonstrated for the first time, that the CGRP receptor is also involved in mechanical force transduction. αCGRP-associated effects are known to be involved and to be indispensable in skeletal adaptation to load; these effects are mainly attributed to sensory nerve-derived αCGRP [19,34]. As a novelty finding, we show that macrophages respond to mechanical load with alterations in αCGRP neuropeptide and receptor expression, but the effect on the musculoskeletal system needs further examination. The αCGRP-dependent increase of *CRLR*, but not *Ramp1*, might lead to an enhanced expression of CRLR bound to Ramp2 or 3, the receptor for adrenomedullin [35] and thus induce a shift in neuropeptide responsiveness.

Cyclic stretch caused an increase in caspase 3/7 mediated apoptosis in RAW264.7 cells as well as in primary BMM derived from mice 8 weeks after DMM surgery, but proliferation remained unaffected. We assumed that BMM from OA animals react differently to mechanical loading in the context of neuropeptide stimulation because OA is a pathophysiology associated with altered biomechanics [36]. In vivo, increasing apoptosis of macrophages after loading might prevent an overactivation of macrophages responding to load-induced microenvironmental damages thereby preventing an innate immune response. This would be particularly beneficial in pathologies like OA characterized by increased structural damage and release of matrix components. Besides, we showed previously that rat BMM differed in adhesion, proliferation and their reactivity to sympathetic neurotransmitter stimulation after the induction of collagen-induced arthritis [37]. In general, mechano-response of macrophages was associated with increased proliferation [38,39] but especially in C2C12 myoblasts and vascular smooth muscle cells, studies showed highly variable effects of cyclic stretch on proliferation and apoptosis. [40,41]. Furthermore, the stretch was reported to upregulate the gene expression of adhesion molecules in periodontal ligament cells [42], which was not the case in our study. SP application during loading provoked a strong reduction in adhesion, but also affected unloaded RAW cells in a concentration-dependent way. Recent studies reported that SP increased adhesion, e.g., in costal chondrocytes [43], enhanced expression of adhesion-associated genes, e.g., in rat fibroblasts and non-human primate fibroblasts [44] and reorganized the actin skeleton as well as induced focal adhesion formation in keratocytes [45]. Macrophages mostly use different subsets of integrins and scavenger receptors for adhesion that differ also in between macrophage species [3] thus being a possible explanation for the divergent effects of SP stimulation in our system. 

In the context of neuropeptide stimulation, we observed only marginal effects on proliferation and apoptosis of RAW264.7 macrophages and BMM from OA and control animals, contrasting other studies which describe SP as pro-proliferative [31,43,46] and anti-apoptotic in, e.g., tenocytes and pre-adipocytes [47,48]. Support comes from studies of our group, where we showed that proliferation of BMM isolated from mice deficient for SP was not altered [21] and that costal chondrocyte apoptosis remained unaffected by SP stimulation [43]. Both SP and αCGRP did not affect proliferation.

An important feature of macrophages is their phenotype plasticity, which determines macrophage function and requires tight control to avoid deleterious effects that might lead to maladaptation of immune reactions. It was reported before that SP, as well as a mechanical force, could affect macrophage polarization. SP promoted the M2 subtype [28,49,50] whereas mechanical stretch, depending on the intensity, induced both subtypes, M1 and M2 [3]. No comparable studies exist for αCGRP effects, but it was described as a co-stimulatory mediator in LPS-activated macrophages inducing a regulatory, IL-10 producing phenotype [51]. Loading alone induced the upregulation of mRNA for *TNFα*, *iNOS* and *IL-6* indicating a preference of M1 macrophages after 10% cyclic stretching in RAW macrophages. SP stimulation increased the gene expression of the M2 marker *mannose receptor 1*, but decreased *KLF4* and had no additional effects after loading. Due to the high heterogeneity, not all M2 macrophages need to express all markers associated with this phenotype. Along this line, our results indicate the induction of a certain M2 subtype by SP. 

Potentially, the influence of sensory neuropeptides on macrophage phenotype and metabolic activities might be increased by stimulation with higher neuropeptide concentrations. At least for SP, it was reported that macrophages respond better to micromolar concentrations than to nanomolar concentrations [52] used in our study. If this also applies for αCGRP is unknown but should be elucidated in future studies. 

Independent from the macrophage phenotype, expression of ROS is a characteristic feature of both macrophage subtypes, but with different functional aspects. Cyclic stretch-induced ROS release from osteoblast-like cells [53]. Oppositely, the induction of an anti-oxidant response has been demonstrated in mesenchymal stem cells by Chen and co-workers lately [54]. In RAW macrophages, we observed no effect of cyclic stretch on ROS expression. Additional stimulation with SP or αCGRP initially enhanced ROS activity after stretch, but suppressed ROS after prolonged stimulation. In accordance, SP was shown to inhibit apoptosis by the NK1R-dependent scavenging of ROS in corneal epithelial cells [55] similar to αCGRP that, e.g., inhibited hyperoxia-induced ROS production in alveolar epithelial cells [56]. The inhibitory effect on ROS production might result from changes in expression of NK1R and CRLR we observed after loading, but so far the precise underlying mechanisms remain inconclusive. 

In general, cyclic loading increased the sensitivity of murine macrophages to SP and αCGRP stimulation, especially regarding adhesion and ROS activity. Whether these effects are beneficial or deleterious in vivo remains elusive, but could indicate the higher mobility and the activity of macrophages in musculoskeletal tissues subjected to loading (Figure 8B).

Furthermore, macrophages display intense regulation of osteoclast differentiation by mechanical signals and sensory neuropeptide stimulation where SP acts opposite to αCGRP in enhancing osteoclast differentiation and activity [29,57]. Short term mechanical stress by three-point bending inhibited osteoclast differentiation [58] but the cyclic stretch, like we used in our system, increased the number of osteoclasts and resorption area in a different study [59]. We hypothesized that osteoclastogenesis after loading would be different in BMM between OA and control mice but the data were inconclusive. RAW macrophages upregulated *rank* mRNA after loading but the increase was below the limit of physiological effectiveness. *CSF1R* mRNA expression remained unaffected in our study opposite to a study from Yang et al. who showed an increase of the MCSF receptor mRNA after the application of cyclic stretch [60]. SP stimulation decreased *rank* and *CSF1R* mRNA in unloaded cells, whereas αCGRP only decreased CSF1R mRNA. The loaded cells did not respond to SP and αCGRP the same way, maybe due to changes in intracellular signaling similar to observations in pathophysiological conditions [32,61].

## 4. Materials and Methods

### 4.1. RAW 264.7 Cells and Cell Culture

RAW264.7 cells (ATCC-Nr. TIB-71, passage 8) were a kind gift from Prof. Anita Ignatius from the University of Ulm. Cells were used from passage 9 to 15 and were cultured in Dulbeccos Minimal Essential Medium (DMEM, Gibco, #11971-025, Life Technologies, Darmstadt, Germany) supplemented with 10% fetal calf serum (FCS), 2% GlutaMAXTM-I (100×, #35050-38, Gibco, Life Technologies, Darmstadt, Germany) and 1% antibiotics/antimycotics (Sigma, Taufkirchen, Germany) in an incubator set to 37 °C and 5% CO_2_. 

### 4.2. Mechanical Loading

For the application of mechanical loading, we used a custom-made device from the Department of Orthodontics (University Hospital Regensburg) that was constructed similarly to the tension system from the Flexcell International Corporations^®^. The device was built in cooperation with the Department of Physics from the University of Regensburg and uses a stamp that is pushed against a flexible membrane, thereby stretching the membrane and extending this mechanical stretch to cells seeded onto this membrane. The system was constructed using six stamps to apply stretch to 6-well plates with a flexible bottom. For all experiments, we used untreated 6-well BioFlex^®^ Culture plates (#BF3001-U, Flexcell Int. Corporations, Burlington, NC, USA) except for osteoclastogenesis assays of bone marrow-derived macrophages (BMM) that required collagen type I-coated BioFlex^®^ culture plates (#BF-3001C). Measurements assured an equally distributed biaxial stretch along the membrane except for the outer margins. The device was kept in an incubator set to 37 °C and 5% CO_2_. Plates were set to a pre-tension of 110 µC and were stretched with a frequency of 1 Hz and an amplitude of 10% stretch. A single stress session was applied for 4 h per day, and was repeated for two to five consecutive days, depending on the experimental setting. Afterward, cells were harvested and used for the respective assays.

### 4.3. Animals

For the induction of OA, 8–10 weeks old male C57Bl/6J (WT) mice were purchased from Charles River Laboratories (Sulzfeld, Germany) and were adapted to standard housing conditions under a 12 h dark/light cycle until the age of 12 weeks. The mice had access to food and water ad libitum. All animal experiments were approved by the ethical committee of the local authorities (Regierung Unterfranken, AZ 55.2-2531-2-289, date of approval 27 July 2016).

### 4.4. Destabilization of the Medial Meniscus (DMM)

Osteoarthritis was induced following a method described by Glasson [62]. Briefly, after intraperitoneal anesthesia with fentanyl, medetomidin and midazolam, a 3-mm skin incision was made between the distal patella and the proximal tibia plateau of the right leg, exposing the knee joint. The joint capsule was opened with a 1–2 mm incision medial to the patellar tendon. For induction of OA, the medial meniscotibial ligament was dissected carefully after visualization using microscissors. Sham surgery was performed in the right knee of the control group with visualization of the ligament only. The joint capsule and skin were closed and animals received analgesia (buprenorphine in 0.9% NaCl solution, 0.1 mg/g body weight). Immediately after surgery, animals were allowed to move freely and recover from the surgery. After 2 and 8 weeks, animals were asphyxiated with CO_2_ followed by cervical dislocation and the bone marrow was isolated.

### 4.5. Isolation of Bone Marrow-derived Macrophages

BMM were isolated 2 and 8 weeks after either DMM or Sham surgery as described by Niedermair et al. [21]. Briefly, the tibia and femur of the operated right leg were isolated and the distal ends of both bones were cut off. The bone marrow was rinsed out and after centrifugation, red blood cells were lysed via hypotonic shock using cold Aqua bidest. Physiological salt concentrations were restored using 10× PBS. Cells were centrifuged and the pellet was resuspended in α−Minimal Essential Medium (αMEM, Sigma, Taufkirchen, Germany) containing 10% FCS, 2% GlutaMAXTM-I (100×, #35050-38, Gibco, Life Technologies, Darmstadt, Germany) and 1% antibiotics/antimycotics (Sigma, Taufkirchen, Germany) as well as 20 ng/mL murine M-CSF (#315-02, PeproTech, Hamburg, Germany) as a macrophage survival factor. Cells were seeded into bacterial Petri dishes and cultivated for 3 days. Adherent macrophages were detached with cold 0.02% EDTA/ PBS and cells were subjected to mechanical stress and, afterward, seeded into the respective assays. 

### 4.6. RNA Isolation and cDNA Synthesis

One million RAW264.7 cells were seeded into each cavity of a 6-well BioFlex^®^ culture plate (untreated, #BF3001-U, Flexcell Int. Corporations, Burlington, NC, USA). Cells were collected for RNA isolation after 4-h loading sessions per day on two consecutive days. Cells were lifted from the plate, pelleted and stored at −80 °C until further use. RNA was isolated using the Absolutely RNA Miniprep Kit (Agilent Technologies, Santa Clara, CA, USA) according to the manufacturer instructions. The RNA quantity was measured using the Nanodrop 1000 spectrometer (Thermo Scientific, Waltham, MA, USA) and the RNA quality was assured at random using the 2100 Bioanalyzer from Agilent Technologies (Santa Clara, CA, USA). RNA was transcribed into single-stranded cDNA with the AffinityScript QPCR cDNA Synthesis Kit from Agilent Technologies (Santa Clara, CA, USA) using oligo and random primers.

### 4.7. Quantitative Real-time PCR

Influence of loading on relative gene expression of genes related to osteoclastogenesis, macrophage polarization and sensory neuropeptides and the respective receptors (primer listed in Table 1 were purchased from MWG Eurofins, Ebersberg, Germany or Microsynth AG, Balgach, Switzerland) were analyzed using the Mx3005P QPR System from Agilent Technologies (Santa Clara, CA, USA) with the Brilliant II SYBR Green QPCR Master Mix with ROX (Agilent Technologies, Santa Clara, CA, USA). Expression of the gene of interest was normalized to the expression of the endogenous control gene *glyceraldehyde 3-phosphate dehydrogenase* (*GAPDH*) and relative gene expression of loaded cells relative to the gene expression of unloaded cells was determined with the ΔΔ*C*t method.

### 4.8. Stimulation of RAW264.7 and BMM with SP and αCGRP and Receptor Antagonists

For all experiments, RAW264.7 and BMM were stimulated with 10^−8^ and 10^−10^ M of recombinant SP (#S6883, Sigma-Aldrich, Schnelldorf, Germany) or mouse/rat αCGRP (#4025897, Bachem, Bubendorf, Switzerland). BMM experiments also included a neuropeptide combination with 10^−7^ M of a respective receptor antagonist: for SP the NK1R antagonist L733,060 (#1145, Tocris, Biotechne, Minneapolis, USA) and for αCGRP the antagonist CGRP_8–37_ (#4034544, Bachem, Bubendorf, CH). For RNA extraction, RAW cells were stimulated during loading. For cell assays such as adhesion, proliferation, apoptosis and ROS activity, RAW cells were either stimulated during loading (pre), during assay procedure (post) or constantly throughout the experiment (constant). BMM experiments were only stimulated during the assay procedure.

### 4.9. Caspase 3/7 Apoptosis Assay

Apoptosis induction was measured using the Apo-ONE^®^ Homogenous Caspase-3/7 Assay from Promega (#G7791, Madison, WI, USA). Briefly, RAW264.7 or BMM were harvested from the untreated Bioflex plates (Flexcell corp.) after loading, were counted and seeded in black 96-well plates with a clear bottom. The assay was performed according to the manufacturer instructions and fluorescence was measured at 485 nm after 2, 6, and 24 h. The assay was performed in triplicates in at least five (RAW cells) or three (BMM) independent experiments.

### 4.10. BrdU Incorporation Proliferation Assay

The proliferation of RAW264.7 and BMM after 2 days of loading was analyzed using the colorimetric BrdU cell proliferation ELISA from Roche (#11647229001, Basel, CH). Briefly, RAW264.7 or BMM were harvested from the untreated Bioflex plates (Flexcell corp.) after loading, were counted and seeded in clear 96-well plates. Cells were allowed to recover overnight and synchronized by serum deprivation for 24 h. After 24 h, BrdU labeling reagent was added, with or without additional stimulation, and cells were cultured for an additional 48 h. Afterward, cells were fixed and processed according to the manufacturer instructions. The assay was performed in triplicates in at least five (RAW cells) or three (BMM) independent experiments.

### 4.11. Crystal Violet Adhesion Assay

To analyze the influence of mechanical stress on adhesion capacity, RAW264.7 cells were harvested from untreated Bioflex plates (Flexcell corp.) after 2 days of loading and 30,000 cells per well were seeded in a clear 96-well plate with or without neuropeptide stimulation. After 20 min, the wells were washed to remove non-adherent cells and adherent cells were fixed with 1% glutaraldehyde. Fixed cells were stained with a 0.02% crystal violet solution for 15 min and thoroughly washed afterwards. The amount of incorporated crystal violet dye is considered proportional to the number of cells attached in each well. Incubation in 70% ethanol for 1 h on an orbital shaker dissolved crystal violet out of the cells. Absorption of ethanol-dissolved crystal violet dye was measured by using a microplate reader (Tecan) at 595 nm. The assay was performed in triplicates in at least five independent experiments.

### 4.12. ROS Activity Assay

ROS activity was measured using the Reactive Oxygen Species (ROS) Detection Kit from Promocell (#PK-CA577-K936, Heidelberg, Germany). Briefly, RAW264.7 cells were harvested from the untreated Bioflex plates (Flexcell corp.) after loading, were counted and seeded in black 96-well plates with a clear bottom. The assay was performed according to the manufacturer instructions and fluorescence was measured at 495 nm after 1 and 24 h. The assay was performed in triplicates in at least five independent experiments.

### 4.13. Protein Expression of Sensory Neuropeptide Receptors in RAW264.7 Cells

The protein expression of the receptors for SP (NK1R) or αCGRP (CRLR, Ramp1) was analyzed in cell pellets harvested after 1, 2 or 3 days of mechanical loading via Western blot. Therefore RAW264.7 cells were seeded in untreated 6-well Bioflex plates (Flexcell corp.) and either subjected to a single 4-h loading session or for 2 or 3 sessions respectively. Immediately after loading, cells were lysed in RIPA buffer containing a protease inhibitor cocktail (CompleteMini, Roche, Basel, CH). Protein lysate was stored at −20 °C until further analysis. The protein content was measured using the Pierce BCA protein assay kit (#23227, Thermo Scientific, Waltham, MA, USA) according to the manufacturer instructions. Samples were denatured for 5 min at 95 °C and 40 µg of total protein loaded onto a 12% SDS gel. Separated proteins were transferred onto a 0.45 µm nitrocellulose membrane via tank blot procedure. Unspecific binding sites were blocked with 5% dry milk (Carl Roth, Karlsruhe, Germany) dissolved in Tris-buffered-saline with Tween 20 (T-TBS), for 1 h at room temperature. Primary antibodies rabbit anti-neurokinin receptor 1 (1:20.000, monoclonal, Abcam, #183713, Cambridge, UK), rabbit anti-calcitonin receptor-like receptor (CRLR, 1:500, polyclonal, Bioss antibodies, #bs-1860R, Boston, MA, USA) or rabbit anti-receptor activity modifying protein 1 (Ramp1, 1:5000, #ab156575, abcam, Cambridge, UK) were diluted in 5% dry milk/T-TBS and incubated on a shaker at 4 °C overnight. As an endogenous loading control, β-actin was detected in parallel (1:5.000, monoclonal, Abcam, #ab8227). Primary antibodies were detected using the donkey anti-rabbit peroxidase-coupled polyclonal antibody from Jackson ImmunoResearch (1:10.000, #711-036-152, Cambridgeshire, UK). For signal detection, membranes were incubated in an ECL substrate solution from the Pierce ECL Western Blotting Substrate Kit (NK1R and Ramp1, Thermo Scientific, Waltham, MA, USA) or Pierce SuperSignal West Femto Kit (CRLR, Thermo Scientific, Waltham, MA, USA). Membranes were developed either using the Chemi smart 500 chemiluminescence lamp from PeqLab (NK1R and CRLR, Erlangen, Germany) or the GelDoc Imager from Biorad (Ramp1). Receptor signals were evaluated densitometrically using Adobe Photoshop relative to the expression of β-actin as the endogenous loading control.

### 4.14. Production of Endogenous SP and αCGRP

To evaluate neuropeptide production after loading, RAW264.7 were seeded into untreated 6-well Bioflex plates (Flexcell corp.) and subjected to 1, 2 or 3, 4-h-loading sessions on respective consecutive days. After the last loading session, the medium was exchanged for FCS-free medium and cells were incubated for an additional 24 h to facilitate protein production. Afterward, the culture supernatant was collected and mixed with protease inhibitor cocktail (CompleteMini, Roche) and stored at −20 °C until further analysis. For detection of SP, the Substance P ELISA kit was purchased from Enzo Life Sciences Inc. (#ADI-900-018, Farmingdale, NJ, USA) and for the detection of αCGRP the rat/mouse, the “Enzyme Immunoassay Kit” from Phoenix Pharmaceutical Inc. (#EK-015-09, Burlinghame, CA, USA) was used. Both ELISA kits were used according to the manufacturer instructions.

### 4.15. Osteoclastogenesis of BMM during Mechanical Loading

Osteoclastogenesis was induced in BMM cells under the application of mechanical loading. Cells were seeded into 6-well Flexwell plates coated with collagen type 1 (#BF-3001C, Flexcell Int. Corporations, Burlington, NC, USA) in a BMM medium supplemented with 20 ng/mL murine M-CSF (#315-02, PeproTech, Hamburg, Germany) and 50 ng/mL murine Rankl (#462-TEC/CF, R&D Systems, Biotechne, Minneapolis, USA) to induce osteoclastogenesis. After seeding, cells were subjected to a mechanical load for 4 h/day on 5 consecutive days. Subsequently, cells were fixed and stained for tartrate-resistant acid phosphatase activity using the Acid Phosphatase, Leukocyte (TRAP) kit from Sigma-Aldrich (#A387, Taufkirchen, Germany). Membranes of the plates were scanned using the TissueFaxs system. Cells containing three or more nuclei were considered as osteoclasts and were counted.

### 4.16. Statistical Analysis

Statistical analysis of the data was performed using GraphPad Prism 5 and 6. For comparison of non-transformed data (when control was not set to 100%), non-parametric Mann-Whitney test was used. Comparisons of stimulation effects against unstimulated control conditions set to 100% were analyzed using the one sample *t*-test. To evaluate the effect of loading, controls without loading were set to 100%. Results are presented as box plots with the interquartile range and whiskers from minimum to maximum. Osteoclast numbers are presented as bars representing the mean + standard error of the mean (SEM). *P*-values below 0.05 were considered statistically significant. 

## 5. Conclusions

In this study, we demonstrated that SP and its receptor NK1R, as well as αCGRP and its receptor CRLR/Ramp1, are involved in the mechano-regulation of murine macrophages. Especially the involvement of the αCGRP system in mechano-regulation has not been addressed before. Because mechanical loading and sensory stimulation are involved in the turnover and regeneration of musculoskeletal tissues like bone, understanding its impact on resident macrophages contributes to our understanding of the local cellular interactions that might be involved in physiological and pathophysiological processes. Loading altered the reactivity to SP and αCGRP regarding adhesion and ROS production suggesting mechano-dependent alterations in G-protein receptor signaling that might affect macrophage migration and activity. Furthermore, OA induction altered BMM apoptosis in response to loading indicating that OA-associated biomechanical alterations also affect the bone resident macrophage population. Future studies should examine whether macrophages express an alternative, truncated NK1R isoform [63] or show altered cellular localization of the receptor [32], helping explain the altered macrophage responses to neuropeptide stimulation.

## Figures and Tables

**Table 1:** The primer sequences used for quantitative real-time PCR.

Primer	FORWARD: 5′-3′ SEQUENZ REVERSE: 5′-3′ SEQUENZ	Amplicon Size (bp)
**Neuropeptides and Receptor Genes**		
*NK1R*	Fwd: ATTGAGTGGCCAGAACATCC Rev: ACTGGCCCACAGTGTAATCC	135
*CRLR*	Fwd: GCCAATAACCAGGCCTTAGTG Rev: GCCCATCAGGTAGAGATGGAT	77
*Ramp1*	Fwd: CCTGACTATGGGACTCTCATCC Rev: CGTGCTTGGTGCAGTAAGTG	139
*SP*	Fwd: GATGAAGGAGCTGTCCAAGC Rev: GCACAGGAGTCTCTGCTTCC	102
*CGRP*	Fwd: TGCAGGACTATATGCAGATGAAA Rev: GGATCTCTTCTGAGCAGTGACA	91
**Osteoclastogenesis-Related Genes**		
*CSF1R*	Fwd: CAGAAGACCCACCTTCCAAC Rev: CTGCTTGGCAGGTTAGCATA	93
*Rank*	Fwd: GCTCCTGAAATGTGGACCAT Rev: CACGATGATGTCACCCTTGA	241
**Macrophage Polarization Genes**		
*NFκB*	Fwd: GGCAGCTCTTCTCAAAGCAG Rev: CCACTCCCTCATCTTCTCCA	107
*TNFα*	Fwd: GACAGTGACCTGGACTGTGG Rev: GAGACAGAGGCAACCTGACC	132
*iNOS*	Fwd: CAAGCACCTTGGAAGAGGAG Rev: AAGGCCAAACACAGCATACC	149
*IL-6*	Fwd: CCGGAGAGGAGACTTCACAG Rev: CAGAATTGCCATTGCACAAC	134
*IL-10*	Fwd: CCAAGCCTTATCGGAAATGA Rev: TTTTCACAGGGGAGAAATCG	162
*Mrc1*	Fwd: AGAAAATGCACAAGAGCAAGC Rev: GGAACATGTGTTCTGCGTTG	101
*KLF4*	Fwd: CCGTCCTTCTCCACGTTC Rev: GAGTTCCTCACGCCAACG	93

## Supplementary Materials

Supplementary materials can be found at http://www.mdpi.com/1422-0067/20/3/503/s1.

## Abbreviations

SP	Substance P
αCGRP	alpha-calcitonin gene-related peptide
NK1R	Neurokinin receptor 1
CRLR	Calcitonin receptor-like receptor
Ramp1	Receptor activity-modifying protein 1
BMM	Bone marrow-derived macrophage
OA	Osteoarthritis
DMM	Destabilized medial meniscus
NGF	Nerve growth factor
MHCII	Major histocompatibility complex class II
IL	Interleukin
LPS	Lipopolysaccharide
TNF	Tumor necrosis factor
TRPV1	Transient receptor potential cation channel subfamily V member 1
KLF4	Krüppel-like factor 4
Mrc-1	Mannose receptor 1
iNOS	Inducible NO synthase
CSF1R	Colony stimulating factor 1 receptor
NFκB	Nuclear factor “kappa light-chain enhancer” of activated B cells
PCR	Polymerase chain reaction
