# Supplementary material for: Sensory Neuropeptides and their Receptors Participate in Mechano-Regulation of Murine Macrophages
Source: Int J Mol Sci. 2019 Jan 24;20(3):503. doi: 10.3390/ijms20030503 (PMC6386869; doi:10.3390/ijms20030503)
Supplement: Supplementary file 1 [file ijms-20-00503-s001.pdf]

Supplementary Figures

Sensory neuropeptides and their receptors participate in mechanoregulation of murine macrophages

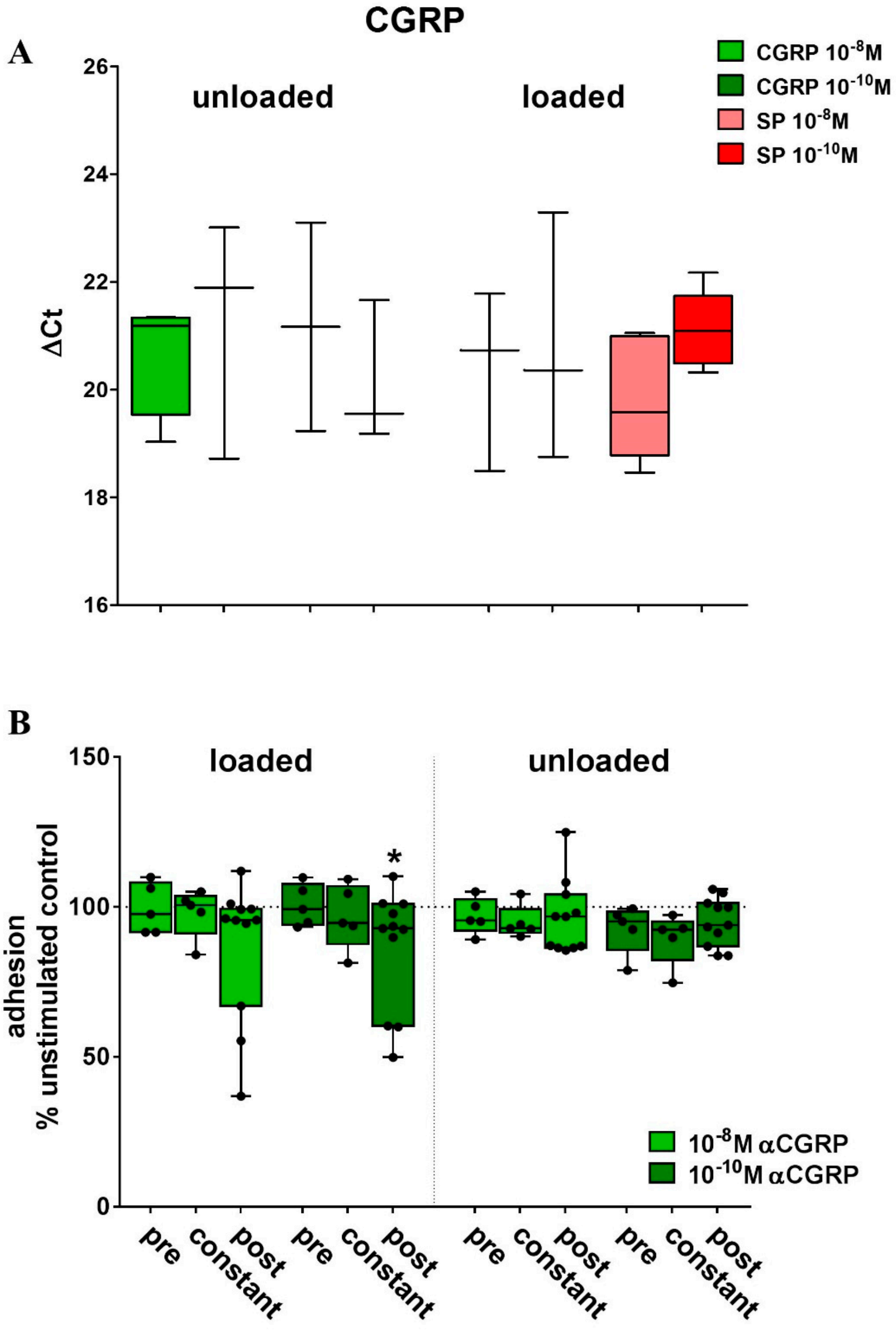

Supplementary figure S1: Influence of neuropeptide stimulation on αCGRP gene expression in RAW264.7 cells. A) Alterations of αCGRP gene expression by stimulation with SP and αCGRP after 4 hour loading per day on two consecutive days was analyzed using quantitative real-time PCR. Results are depicted as ΔCt values. Normalizer: GAPDH. N=5.

B) shows the impact of stimulation with αCGRP on adhesion of loaded and unloaded RAW264.7 in relation to the respective unstimulated cells (=100%). Graphs depict effect of stimulation during loading (pre), during loading and assay (constant) and only during assay procedure (post). Pre, constant N=5, post N=11. One sample t test *p<0,05.

SP – substance P, αCGRP – calcitonin gene-related peptide.

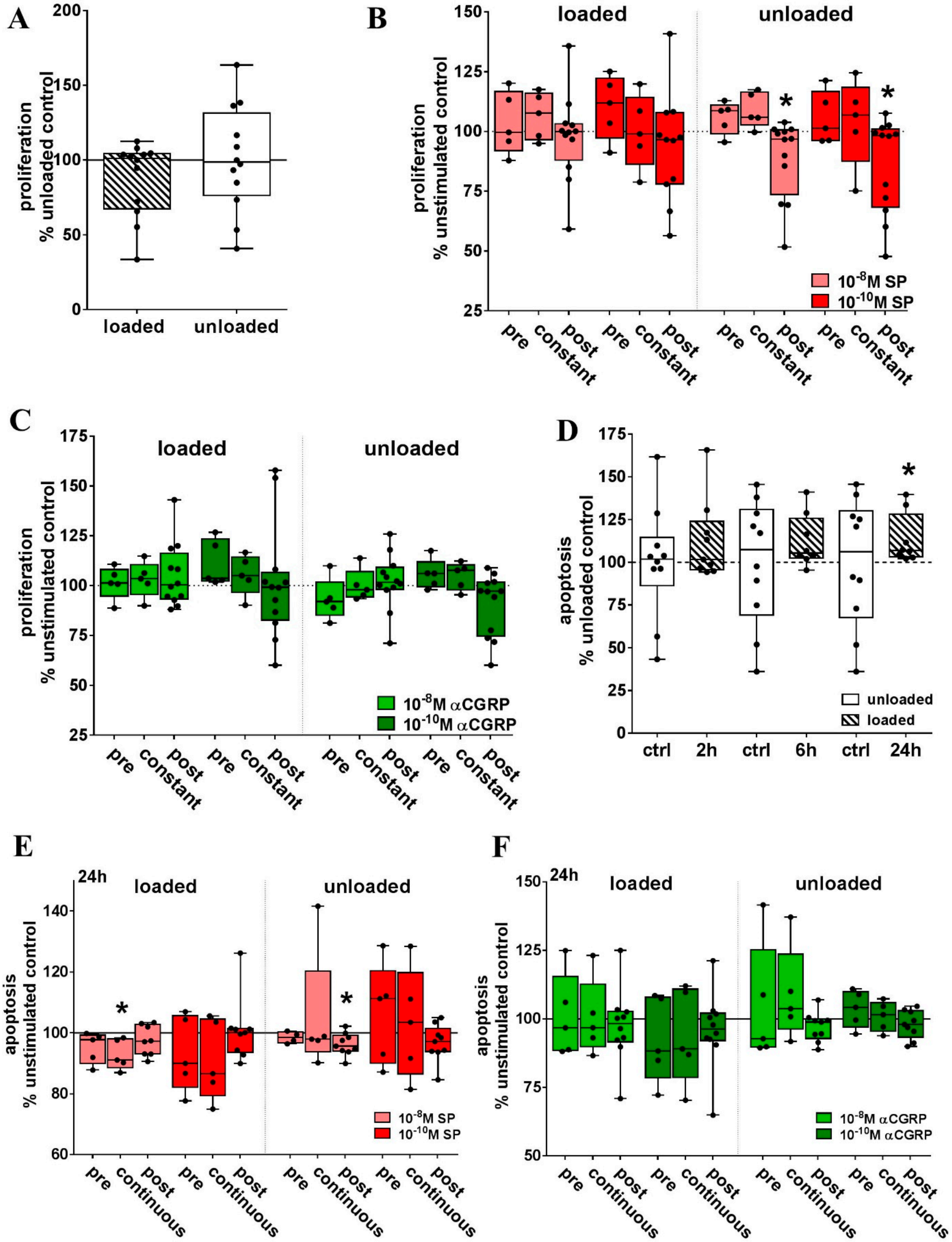

Supplementary figure S2: Influence of sensory neuropeptide stimulation and mechanical loading on proliferation and apoptosis of RAW264.7 cells.

A, D) The graph depicts the proliferation (A) and apoptosis (B) of loaded RAW264.7 cells relative to unloaded RAW264.7 cells (set to 100%). One sample t test *p<0,05. N=10.

B, C) The graphs show the impact of stimulation with SP (B) and αCGRP (C) on proliferation of loaded and unloaded RAW264.7 in relation to the respective unstimulated cells (=100%). Graphs depict effects of stimulation during loading (**pre**), during loading and assay **(constant**) and only during assay procedure (**post**). Pre, constant N=5, post N=11. One sample t test *p<0,05.

E, F) The graphs show the impact of stimulation with SP (E) and αCGRP (F) on apoptosis of loaded and unloaded RAW264.7 in relation to the respective unstimulated cells (=100%). Graphs depict effects of stimulation during loading (**pre**), during loading and assay (**constant**) and only during assay procedure (**post**). Pre, constant N=5, post N=11. One sample t test *p<0,05.

SP – substance P, αCGRP – alpha-calcitonin gene-related peptide

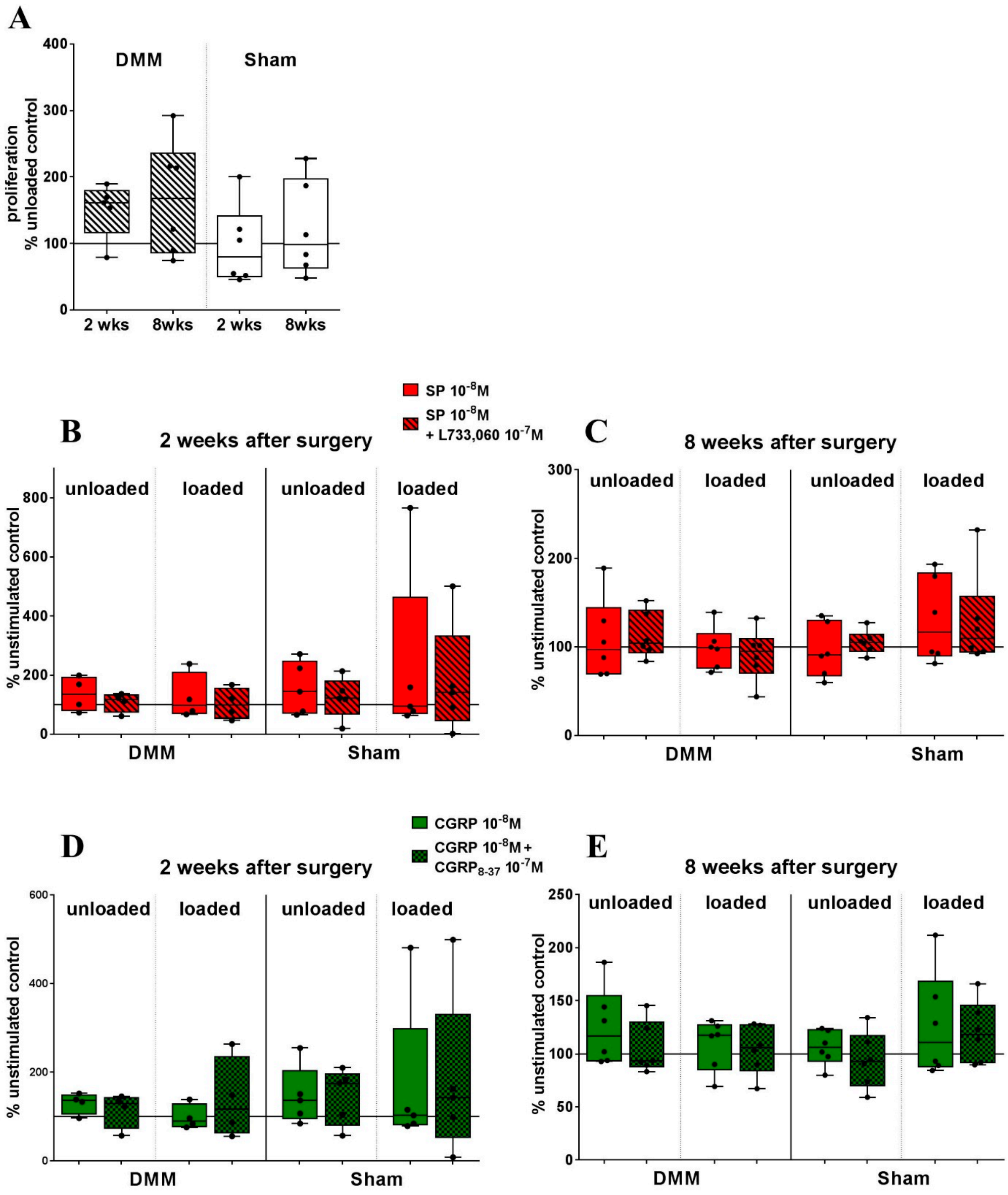

Supplementary Figure S3: Influence of sensory neuropeptides on proliferation of BMM from DMM and Sham mice after mechanical loading

A) Analysis of proliferative capacity of BMM isolated 2 and 8 weeks after DMM or Sham surgery and subjected to 4 hour loading per day on 2 consecutive days depicted in relation to proliferation of unloaded cells (=100%). N=5-6. One sample t test *p<0,05.

B-E) Effect of stimulation with SP or SP combined with the NK1R antagonist L733,060 (B, C) or αCGRP and αCGRP combined with the αCGRP receptor antagonist $CGRP_{8-37}$ (D, E) on proliferation of BMM isolated 2 (A, C) and 8 (B, D) weeks after DMM or Sham surgery in relation to unstimulated cells (=100%). N=5-6.

BMM – bone marrow-derived macrophages, DMM – destabilized medial meniscus, SP – substance P, αCGRP – alpha-calcitonin gene-related peptide, NK1R – neurokinin receptor 1.

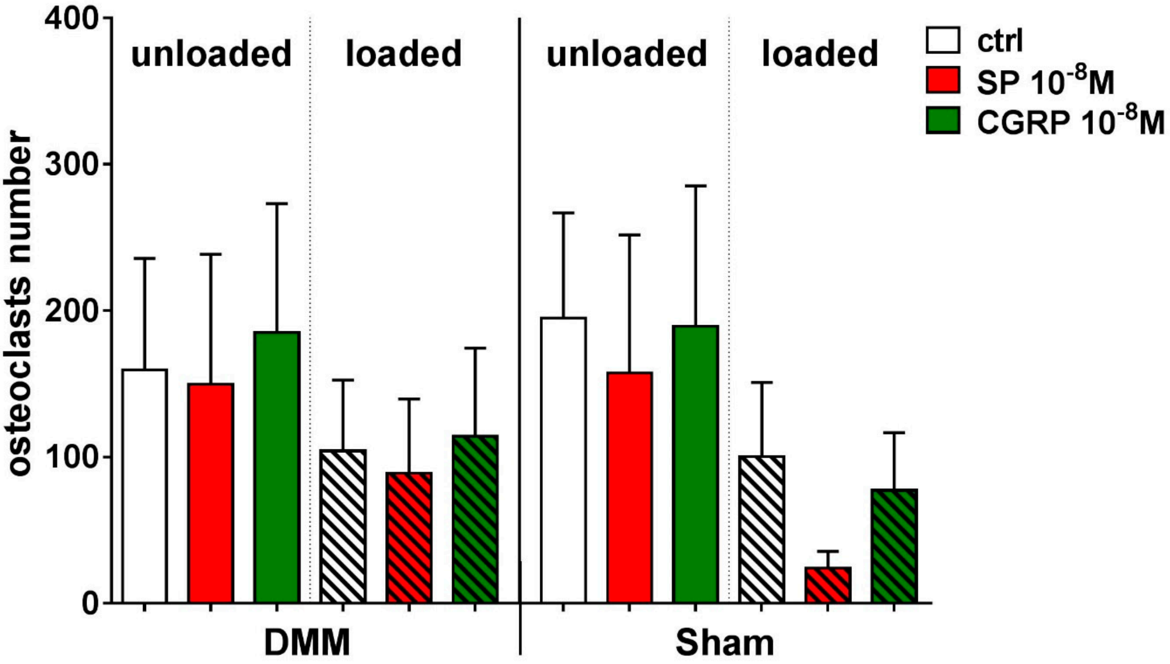

Supplementary figure S4: Influence of mechanical loading and sensory neuropeptide stimulation on osteoclastogenesis of BMM isolated 8 weeks after DMM or Sham surgery.

BMM were isolated 8 weeks after DMM or Sham surgery and subjected to osteoclastogenesis under loading. The graph shows absolute osteoclast numbers per well of unstimulated BMM and BMM stimulated with 10^{-8}M SP or 10^{-8}M αCGRP that remained unloaded or were loaded for 4 hours / day for 5 consecutive days. N=5-6.

BMM – bone marrow-derived macrophage, DMM - destabilized medial meniscus, SP – substance P, αCGRP – calcitonin gene-related peptide
